# Telerehabilitation for a Non-specific Low Back Pain: A Case Report

**DOI:** 10.7759/cureus.47854

**Published:** 2023-10-28

**Authors:** Priti Mehendale, Madhavan Iyenagar, Geeta D Bhatt, Khyati Kothary

**Affiliations:** 1 Department of Musculoskeletal Physiotherapy, K J Somaiya College of Physiotherapy, Mumbai, IND; 2 Department of Physiotherapy, Parul University, Vadodara, IND; 3 Department of Surgery, Parul Sevashram Hospital, Parul Institute of Medical Sciences and Research, Vadodara, IND; 4 Department of Neurophysiotherapy, K J Somaiya College of Physiotherapy, Mumbai, IND; 5 Department of Cardiorespiratory Physiotherapy, K J Somaiya College of Physiotherapy, Mumbai, IND

**Keywords:** smartphone application, virtual physiotherapeutic rehabilitation, kinesiophobia, non-specific low back pain, telerehabilitation

## Abstract

One of the most common conditions that affect activities of daily living and make them significantly more difficult to perform is low back pain (LBP). As a result, it is essential to treat LBP at an early stage. Particularly in geographically remote areas where there is a shortage of medical professionals and a lack of rehabilitation services, telerehabilitation is considered a potential alternative. Hence, this case report represents the impact of telerehabilitation on LBP in a 32-year-old female corporate worker who presented to the out-patient department of physiotherapy with the chief complaints of LBP for the last three months with difficulty in performing activities, and being unable to sit for prolonged period of time. The physiotherapeutic rehabilitation was virtually administered through online sessions through the cloud-based application as the patient was not able to visit the outpatient department on a regular basis. Post-intervention results demonstrated increased range of motion and flexibility, reduced pain, increased muscle strength, reduced disability and kinesiophobia, and improved quality of life. Hence, it can be concluded that telerehabilitation offers a novel solution to increase access to rehabilitation services.

## Introduction

The lower back region involves the lumbar and sacral regions and about 50 per cent of the population at least once a year experiences one of the most prevalent conditions that is low back pain (LBP) [1]. Activities required for daily living are impacted by LBP and become much more challenging to perform. Depending on the area affected, LBP can be at the lumbar, sacroiliac, or lumbosacral level [2]. It can also be categorised into specific (LBP due to certain diseases or structural problems in the spine, or when the pain radiates from another part of the body) or non-specific depending on the cause (LBP with no known underlying pathology) [3]. Depending on how long it lasts, LBP can be classified as acute if it ranges from 0 to 14 days, subacute if ranges from 2 to 12 weeks, or chronic if lasting for more than three months [3-5]. LBP should be treated at an early stage because it will eventually result in greater biomechanical changes which involve reduced range of motion and flexibility of muscles, and poor posture [2].

Patients encounter a number of obstacles, including a gap in service delivery, particularly in geographically remote areas where there is a shortage of medical professionals and limited access to physical therapy rehabilitation services. Hence, telerehabilitation is being considered as a potential means of bridging this gap [6]. Telerehabilitation is defined as the delivery of clinical information and healthcare services remotely using wireless satellite, telephone media, and internet which are information and telecommunication technologies. This will provide widespread access to a range of rehabilitation services which will consequently impact barriers of travel, time, and distance offering benefits to the patient in receiving treatment [7,8]. There are an enormous amount of commercially available programs available for managing and monitoring healthcare with the introduction of smartphones [8]. Hence, this case report demonstrates the impact of telerehabilitation for non-specific LBP.

## Case presentation

A 32-year-old female corporate worker presented to the outpatient department (OPD) of K J Somiaya College of Physiotherapy with chief complaints of LBP for the last three months which was sudden in onset and progressed gradually with difficulty in performing activities, and was unable to sit for a prolonged period of time. The pain aggravated due to prolonged sitting, standing, or while performing a high-intensity activity such as lifting heavy weights during activities of daily living (ADL) because of which she developed kinesiophobia, and the pain was relieved with rest. The patient described a history of prolonged sitting for seven hours per day as a corporate worker and heavy weight lifting while performing ADL. The patient approached an orthopaedician for the same who prescribed pain-relieving medications for seven days but the pain did not subside and therefore was referred to physiotherapy OPD for treatment.

Clinical examination

On palpation, grade 3 tenderness and spasm were present bilaterally in the paraspinal region of the L4-L5 lumbar spine along with tightness of the thoracolumbar fascia and the presence of trigger points in the quadratus lumborum. The pain intensity was 4 at rest and 8 on activity that mainly involved prolonged sitting, standing, or lifting heavy weights while performing ADL which was recorded using the numerical pain rating scale (NPRS). Additionally, the range of motion (ROM) while performing lumbar extension and side rotations was extremely painful and limited. No significant findings were present related to gait and posture. According to the manual muscle testing (MMT), the muscular strength of the lumbar flexors was grade 3+ and for lumbar extensors and side rotators was grade 3. Furthermore, for disability assessment, the Roland Morris Disability Questionnaire [9] was used to assess the functional limitations and the score was 15/24. similarly, kinesiophobia was evaluated using the Tampa scale (TSK-11) [10] on which the score was 35/44, and quality of life was determined using the World Health Organization Quality of Life Brief Version (WHOQOL-BREF) questionnaire [11] on which the score was 62/100.

Therapeutic intervention

The physiotherapeutic rehabilitation was virtually administered through online sessions through the cloud-based application as the patient was not able to visit the OPD regularly. The main aim of the treatment was to reduce pain, improve lumbar ROM, decrease kinesiophobia and functional disability, and improve quality of life. The patient was informed about the protocol and consent was obtained before therapy. The physiotherapeutic intervention was performed for two sessions per week for five weeks, summating into ten sessions over five weeks and each session was carried out for 45 minutes under the guidance of the therapist.

The physiotherapeutic regimen included a back school program which consisted of patient education that involved explanation of relevant anatomy, physiology, pathophysiology, and pathomechanics of the lumbar spine, concept of LBP leading to understanding the causes and factors involved in chronic LBP, postural re-education consisting of understanding of good posture and its benefits, how to attain and maintain good posture, and ergonomics involving education regarding correct techniques of sitting, standing, pulling, pushing, lifting, kneeling, turning, twisting, and transitions [12]. Additionally, to relieve pain heating pad for 10 minutes before and after the intervention was given to the patient.

The exercises performed included a set of back and abdominal strengthening exercises [13] that were given for 10 repetitions with a five-second hold which involved statics, pelvic bridging, pelvic tilts, unilateral pelvic bridging, abdominal curl-ups, sit-ups, and squats [13]. Lower limb flexibility exercises for the hamstrings, iliopsoas, dorso lumbar fascia, quadriceps, and calf muscles which consisted of a stretching duration of 10 seconds with 3 seconds of rest intervals between each stretch, and a total number of three stretches were performed that helped in improving flexibility. Additionally, at the end of each exercise session relaxation was induced through shavasana. All the exercises were demonstrated by the therapist through online sessions along with the progression involved accordingly. The patient was reassessed after five weeks.

Follow-up and outcome measures

After the termination of the treatment, the pain intensity score reported was 2. The patient in total completed five weeks of sessions after which positive outcomes were demonstrated from the treatment, as there was an increase in ROM and flexibility, a reduction in pain, an increase in muscle strength, reduced disability and kinesiophobia, and an improved quality of life as shown in Table 1.

**Table 1:** Pre- and post-comparison of outcome measures TSK-11 = Tampa Scale of Kinesiophobia, WHOQOL-BREF = World Health Organization Quality of Life Brief Version, NPRS = Numerical Pain Rating Scale, MMT = manual muscle testing

Sr. No.	Outcomes	Pre-treatment values	Post-treatment values after five weeks
1.	Pain intensity on NPRS	8 on activity	2 on activity
2.	Lumbar range of motion using modified-modified Schober’s test		
2a.	Flexion	4.1 centimeter	6.2 centimeter
2b.	Extension	1 centimeter	2.2 centimeter
3.	Manual muscle testing (MMT)		
3a.	Flexors	Grade 3+	Grade 4
3b.	Extensors	Grade 3	Grade 4
3c.	Left and right side rotators	Grade 3	Grade 4
4	Roland Morris Disability Questionnaire: 24-item score	15/24	4/24
5.	Quality of life (WHOQOL-BREF) score	62/100	88/100
6	Kinesiophobia through Tampa scale (TSK-11)	35/44	19/44

## Discussion

This case report represents a case of a 32-year-old female with non-specific LBP. The main purpose of telerehabilitation was to reduce pain, increase ROM and flexibility, increase muscle strength, decrease disability and kinesiophobia, and increase quality of life. Telerehabilitation was virtually administered through the cloud-based application as the patient was not able to visit the OPD regularly. Telerehabilitation involves smartphones, telemonitoring, mobile apps, and other similar online tools and devices to inform patients, caregivers, and medical professionals about the disease and provide a collaborative environment to support suggestions and interactions between individuals and those assisting them in managing their disease; and encourage a healthy lifestyle among the general public [8]. There is evidence that these methods work in a variety of patient populations such as with physical disabilities and movement impairment. The positive impact of telerehabilitation is also supported by systematic reviews of the literature [14].

The telerehabilitation regimen involved patient education, postural re-education and ergonomics, back and abdominal strengthening, and lower limb flexibility exercises. A systematic review concluded that a back with more flexibility will have a wider ROM [15]. For maintaining long-term effects, core strengthening exercises were preferred as evidence suggested that it has a positive impact in reducing pain levels. As LBP can reoccur after treatment is discontinued, core stability exercises are an important component during the treatment [16,17].

Numerous studies showed that telerehabilitation can be used as an alternative or as a substitute to regular, institution-based, outpatient, or in-home treatment. In a previous study, over a 24-week period, 48 physical therapy sessions through telerehabilitation were performed from which the patient with LBP experienced positive changes in both cognitive and physical performance. Similarly, an exercise program through a 12-week videoconferencing improved significantly the leg strength and arthritis symptoms of 22 individuals with knee osteoarthritis in Hong Kong community centers [18]. In conformity with American Physical Therapy Association (APTA) guidelines, telemedicine is an appropriate model for the practice of physical therapy, according to a position paper released by the APTA in 2006 [19].

## Conclusions

The case report highlighted a positive impact of telerehabilitation on non-specific LBP as post-intervention results demonstrated increased ROM and flexibility, reduced pain, increased muscle strength, reduced disability and kinesiophobia, and improved quality of life. Telerehabilitation is a novel alternative approach to clinical rehabilitation, but its efficacy as a means of consultation, intervention, and follow-up care needs to be well-documented, investigated, and published. Additionally, various challenges or limitations that were faced involved technical issues, inaccuracy in maintaining appropriate ergonomics while performing exercises, and lack of exercise equipment. Professional groups should establish and disseminate guidelines that will make it simpler to utilize and design telerehabilitation programs to enhance access to a variety of treatments.
